# Sex differences in muscle activity emerge during sustained low‐intensity contractions but not during intermittent low‐intensity contractions

**DOI:** 10.14814/phy2.14398

**Published:** 2020-04-13

**Authors:** Justin J. Kavanagh, Kristen A. Smith, Clare L. Minahan

**Affiliations:** ^1^ Menzies Health Institute Queensland Griffith University Gold Coast Australia; ^2^ Griffith Sports Physiology and Performance School of Allied Health Sciences Griffith University Gold Coast Australia

**Keywords:** central fatigue, gender, peripheral fatigue, steadiness

## Abstract

Sex differences in motor performance may arise depending on the mode of contraction being performed. In particular, contractions that are held for long durations, rather than contractions that are interspersed with rest periods, may induce greater levels of fatigue in men compared to women. The purpose of this study was to examine fatigue responses in a cohort of healthy men (*n* = 7, age [mean] = 21.6 ± [*SD*] 1.1 year) and women (*n* = 7, age: 22.0 ± 2.0 year) during sustained isometric and intermittent isometric contractions. Two contraction protocols were matched for intensity (20% MVC) and total contraction time (600‐s). Biceps brachii EMG and elbow flexion torque steadiness were examined throughout each protocol, and motor nerve stimulation was used to quantify central and peripheral fatigue. Overall, there were few sex‐related differences in the fatigue responses during intermittent contractions. However, men exhibited progressively lower maximal torque generation (39% versus 27% decrease), progressively greater muscle activity (220% versus 144% increase), progressively greater declines in elbow flexion steadiness (354% versus 285% decrease), and progressively greater self‐perception of fatigue (Borg scale: 8.8 ± 1.2 versus 6.3 ± 1.1) throughout the sustained contractions. The mechanism underlying fatigue responses had a muscle component, as voluntary activation of the biceps brachii did not differ between sexes, but the amplitude of resting twitches decreased throughout the sustained contractions (m: 32%, w: 10% decrease). As generating large sustained forces causes a progressive increase in intramuscular pressure and mechanical occlusion—which has the effect of enhancing metabolite accumulation and peripheral fatigue—it is likely that the greater maximal strength of men contributed to their exacerbated levels of fatigue.

## Graphical abstract

This study examined sex‐related neural and muscular mechanisms of fatigue during contraction tasks with and without relaxation phases. Motor performance was similar between men and women when rest phases were available, however women were more resistant to fatigue during low‐intensity steady‐state contractions. The mechanism underlying this difference had an origin in the muscle, as measurements of contractile function differed between sexes, but voluntary activation of the muscle did not differ between sexes.

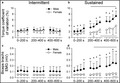

## 1 INTRODUCTION

A common observation made in experiments that assess sex‐related fatigue characteristics is that men are more fatigable than women. This outcome that has been reported for a range of upper and lower limb muscles during low‐to‐moderate intensity intermittent contractions (Ansdell, Thomas, Thomas, Howatson, Hunter, & Goodall, 2017; Hicks & McCartney, 1996; Hunter, Critchlow, Shin, & Enoka, 2004a, 2004b; Maughan, Harmon, Harmon, Leiper, Sale, & Delman, 1986; Pincivero, Coelho, Coelho, & Campy, 2003) as well as sustained isometric contractions (Clark, Collier, Collier, Manini, & Ploutz‐Snyder, 2005; Hunter & Enoka, 2001; Keller et al., 2011). However, there are also reports that motor performance does not differ between sexes when fatigue is induced (Hill et al., 2018; Lee et al., 2017; Mantooth, Mehta, Mehta, Rhee, & Cavuoto, 2018) which highlights the complexity of this topic. Although a body of knowledge is developing regarding how motor performance differs between men and women, the underlying neural, and muscle mechanisms of this performance remain elusive.

There is considerable variation in methodologies that examine sex‐related differences in neural mechanisms of fatigue. However, it appears that the intensity of contraction and the duration of contraction (fixed time versus task failure) may delineate fatigue responses in men and women. For example, while declines in force and neural activation of the muscle are similar for each sex performing repeated submaximal concentric knee extensions, a 60 s maximal isometric contraction of the same muscle group will induce greater levels of central fatigue in men compared to women (Senefeld, Pereira, Pereira, Elliott, Yoon, & Hunter, 2018). In regards to sex‐differences for the upper limb, there is minimal evidence that voluntary drive to the muscle differs at task failure following low‐force or high‐force contractions (Hunter, Butler, Butler, Todd, Gandevia, & Taylor, 2006; Keller et al., 2011; Yoon, Doyel, Doyel, Widule, & Hunter, 2015; Yoon, Schlinder Delap, Schlinder Delap, Griffith, & Hunter, 2007). However, it is curious that men performing low‐force (20% MVC) isometric elbow flexions have consistently shorter times to task failure than females (Yoon et al., 2007, 2015)—a finding that is also evident for contractions of the adductor pollicis (Fulco et al., 1999, 2001). Overall, these findings suggest that measuring voluntary drive to upper limb muscles at absolute time intervals (rather than relative to task failure) during low‐force contractions may provide insight to sex‐related mechanisms of fatigue.

Contraction protocols that include relaxation phases may elicit considerably different responses at the muscle level of men and women compared to sustained steady‐state contractions (Albert, Wrigley, Wrigley, McLean, & Sleivert, 2006; Hunter, Schletty, et al., 2006). However, few upper limb studies match the intensity and duration of different contraction protocols to reveal if sex‐differences emerge for similar workloads. Compared to intermittent contractions, generating sustained muscle forces has the potential to increase intramuscular pressure, increase mechanical occlusion of vasculature supplying the muscle, and decrease muscle perfusion. If these responses are evoked there is potential to exacerbate peripheral fatigue via disturbances in calcium handling, metabolite accumulation, and increased extracellular potassium concentrations (Allen, Lannergren, Lannergren, & Westerblad, 1995; Barcroft & Millen, 1939; Dawson, Gadian, Gadian, & Wilkie, 1980; Sutton, 1992). Although this mechanism is restricted to the muscle, there may also be a neural consequence due to the activation of group III/IV afferents. Stimulation of these muscle afferents can limit the voluntary descending drive to the muscle, or directly inhibit motoneurones at the spinal level, to reduce the development of muscle fatigue (Bigland‐Ritchie, Dawson, Dawson, Johansson, & Lippold, 1986; Blain et al., 2016; Garland, 1991; Woods, Furbush, Furbush, & Bigland‐Ritchie, 1987). Limb occlusion, which is known to activate group III/IV afferents, has been used following fatiguing isometric (30% MVC) handgrip exercise to reveal that mechano‐ and metabo‐reflexes differ between men and women (Ettinger et al., 1996; Minahan et al., 2018; Samora, Incognito, Incognito, & Vianna, 2019). In addition, isometric handgrip exercise differs depending on contraception usage, where normally menstruating women have greater fatigue responses to men compared to women taking hormonal contraception (Minahan et al., 2018). Collectively, this body of evidence implies that sex‐related fatigue responses need to be examined from both a neural perspective as well as a muscle perspective, regardless of contraction modality.

The purpose of this study was to examine fatigue‐related responses in a cohort of healthy young men and normally menstruating women during different modes of muscle contractions. Two elbow flexion protocols were performed where participants performed sustained isometric contractions and intermittent isometric contractions in separate sessions. Tasks in each session were matched for intensity (20% MVC) and total contraction time (600 s). Fatigue‐related changes in biceps brachii electromyography (EMG) and elbow flexion torque steadiness were examined throughout each protocol. Motor nerve stimulation was used to obtain superimposed twitches and resting twitches before, during, and after, each contraction protocol. Collectively, these measurements were used to assess central fatigue and peripheral fatigue that occurred for each mode of contraction for the male and female groups. It was hypothesized that no sex‐related differences would occur during the intermittent contraction protocol. However, we predicted that males would exhibit enhanced characteristics of fatigue during sustained contractions, which would be mediated by an inability to generate force via muscle mechanisms rather than an inability to voluntarily activate via output from the CNS.

## 2 METHODS

### 2.1 Ethical approval

The procedures in this study were performed on humans, where written informed consent was obtained from each participant prior to undertaking any testing procedures. The study received approval from the Griffith University Human Research Ethics Committee, and conformed to the standards set by the Declaration of Helsinki except for registration in a database.

### 2.2 Experiment design and participants

This project was a within‐subjects, two‐way, cross‐over trial. Fourteen healthy participants were recruited for the study. Seven men (age: 21.6 ± 1.1 year) and 7 women (age: 22.0 ± 2.0 year) attended two experimental sessions. In one session participants performed *sustained* 20% MVC isometric elbow flexions, and in the other session, the same subjects performed *intermittent* 20% MVC isometric elbow flexions. The order of the intervention was counterbalanced whereby an equal number of participants performed the sustained contraction and the intermittent contractions in the first testing session.

For inclusion in the study female participants could not be pregnant or using hormonal contraception within the previous 12 months. All women completed their first testing session 2–4 days after the onset of their menstrual period and returned to complete the second session 2–4 days after the onset of their next menstrual period. As such, it was assumed that all women were in the early follicular phase of their menstrual cycle at the time of experimental testing. The timing of the male participant testing sessions was matched so that both groups had similar durations between sessions. All participants were considered recreationally active and met the Australian guidelines for physical activity that includes 150 to 300 min of moderate‐intensity physical activity each week and muscle‐strengthening activities on at least 2 days each week. Participants refrained from any CNS modulators such as caffeine, alcohol, medications, or moderate‐to‐high intensity exercise for 24 hr prior to testing.

### 2.3 Elbow flexion torque and electromyography

**FIGURE 1:**
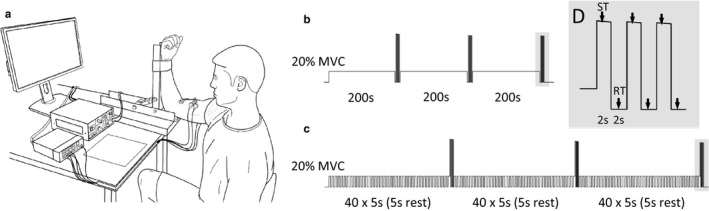
Experimental setup for measurement of elbow flexion torque and elbow flexor muscle activity (a). Participants attended two sessions where isometric elbow flexions were performed at 20% of unfatigued MVC. Sustained isometric contractions were performed in one session (b) and intermittent isometric contractions were performed in the other session (c). The protocols were matched so that the same total contraction time occurred in each session (600 s). For both protocols, 3 maximal contractions (2 s) were performed after the first, second, and third 200 s of total contraction time (shaded areas in b and c, expanded in d). A single electrical stimulus was delivered to the biceps brachii during each MVC so that a superimposed twitch (ST) could be obtained from elbow flexion torque. A single stimulus was also delivered to the muscle during periods of muscle relaxation that occurred between contractions (2 s) so that a potentiated resting twitch (RT) could be obtained from the torque signal

All measurements were performed on the participant's dominant arm using a custom‐designed transducer. An aluminum device positioned the elbow in 90 degrees of flexion, where the participant was seated in a rigid back chair with the humerus horizontal and the forearm oriented vertically (Figure 1a). The arm was secured firmly into the device with a non‐compliant wrist strap so that a PT4000 200kg S‐Type load cell (*PT Ltd*., NZ) attached to the posterior side of the device could measure elbow flexion force. From this load cell data, flexion force could be converted to flexion torque based on the distance between the lateral epicondyle and the point of force application at the wrist. Torque data were sampled at 2 kHz via Spike2 (*CED Ltd*., UK).

Surface EMG was obtained from the biceps brachii and triceps brachii using bipolar 24 mm Ag/AgCl electrodes. Electrodes were aligned in the direction of underlying muscle fibers and had an inter‐electrode distance of 24 mm (*Kendall* ARBO). Surface EMG signals were differentially amplified 1,000 times (NL844, *Digitimer Ltd.*, UK), bandpass filtered with cut‐off frequencies of 10 and 500 Hz (NL135 and NL144, *Digitimer Ltd.*, UK), and then input into a 16‐bit Power 1,401 data acquisition interface (CED Ltd., UK). Surface EMG was sampled via the same Spike2 arrangement used to collect torque data.

### 2.4 Motor point stimulation

Throughout each testing session, electrically evoked increases in elbow flexion torque were measured during maximal contractions (superimposed twitch) and when the muscle was relaxed (resting twitches). The intramuscular nerve fibers innervating the elbow flexors were stimulated with single supramaximal electrical pulses of 100 μs in duration via a constant current nerve stimulator (DS7AH, *Digitimer Ltd.*, UK). A surface anode was placed over the bicipital tendon and a surface cathode was placed on the muscle. Prior to the cathode being affixed, a motor point ball‐pen was used to identify the biceps region that (a) produced the largest twitch response with, (b) the smallest stimulation intensity, and (c) the lowest triceps activation. The stimulation intensity for each participant was set to 20% higher than the intensity required to elicit a maximal resting twitch (range: 55 to 220 mA).

### 2.5 Sustained and intermittent contraction protocols

Each testing session commenced by determining elbow flexion MVC and obtaining baseline twitch measurements. Participants performed 5 brief (~2 s) MVCs where a single electrical stimulus was delivered to the biceps immediately following peak torque of each MVC to obtain a superimposed twitch for the unfatigued muscle. Two seconds after each MVC, a single electrical stimulus was delivered to the resting biceps to obtain a potentiated resting twitch. Rest periods of ~3 min were provided between maximal contractions. The greatest peak torque (not evoked by electrical stimulation) for the 5 trials was used as MVC, and the superimposed twitch and resting twitch from this trial was used as baseline (unfatigued) data.

For both the sustained and intermittent contraction protocols, a target line corresponding to 20% MVC was displayed on a PC monitor directly in front of the participant. A second line that represented elbow flexion torque was also projected on the monitor, where participants were required to regulate their flexion torque to match the target line. As the gain of visual feedback is known to affect force tremor amplitude and targeting error (Kenway, Bisset, Bisset, & Kavanagh, 2014), the scale of the target line y‐axis was normalized between participants and testing sessions so that visual gain was consistent across all conditions. Participants were reminded at the commencement of each submaximal contraction that they had to match the target line to ensure that their attention to the task did not waver.

During the sustained contraction protocol, participants maintained the 20% MVC for a period of 200 s before a series of twitch measurements were made (Figure 1b). After the 200 s contraction, three MVCs (~2 s) were performed with rest periods of 2 s between contractions (Figure 1d). A supramaximal electrical stimulation was delivered to the muscle during each MVC to obtain a superimposed twitch, and after each MVC to obtain a potentiated resting twitch. These procedures were repeated a second and third time so that the total contraction time was 600 s for the session. The intermittent contraction protocol is illustrated in Figure 1c, and followed similar procedures to the sustained contraction protocol. The only difference was that participants performed 40 successive duty cycles comprised of a 5 s contraction and a 5 s relaxation period. Therefore, the protocols were matched so that participants contracted for a total of 600 s—but with different modes of contraction. Participants practiced the contraction protocol at a low‐intensity for ~1 min prior to skin preparation and electrode fixation. This was particularly important for the duty cycle described in the intermittent contraction protocol, as this is a task that few people would be familiar with.

Psychophysical measures of fatigue were assessed using a CR‐10 Borg scale. Ratings of fatigue were collected obtained within the last 5 s of each 200 s contraction window. Participants were instructed that the lowest value on the CR‐10 represented “no fatigue at all,” whereas the highest value on the scale represented “so much fatigue that you would not be able to lift your arm.”

### 2.6 Data analysis

Data were analyzed off‐line using Spike2 software. Data were coded at the commencement of the analysis to ensure that investigators were blinded to the sex of the participant and the mode of contraction. During each contraction protocol the coefficient of variation (CV) was calculated for torque to provide an indicator of steadiness, and the root mean squared amplitude of EMG (EMG rms) was calculated for biceps brachii to determine how the level of muscle activity changed throughout the protocol. Data were analyzed in four equally spaced 5 s bins that corresponded to the same time points in each contraction protocol. Peak torque was calculated from MVCs, and biceps and triceps EMG rms were calculated from a 100 ms window spanning the peak torque (i.e. 50 ms either side of peak). Peak‐to‐peak amplitude of the superimposed and resting twitches was calculated from increases in the torque signal due to electrical stimulation. Time‐to‐peak of the resting twitch was calculated as the duration from the first increase in torque following stimulation to the peak of the twitch. Half‐relaxation time was the duration from peak resting twitch to point corresponding to half of the resting twitch amplitude on the descending limb of the twitch. Voluntary activation (VA) was calculated for baseline measurement and during the contraction protocols, where VA = [1 − (superimposed twitch/resting twitch)] × 100. The superimposed and resting twitches used in the VA calculation were extracted from the same MVC in the contraction protocol.

### 2.7 Statistical analysis

Two sample *t* tests were used to determine if participant characteristics and baseline contraction data differed between men and women. Torque‐derived and EMG‐derived measurements are presented as raw data unless a sex difference was identified at baseline. In this event, the variable has been normalized to its baseline measurement. All torque‐derived measures and all EMG‐derived measures obtained throughout the contraction protocols were examined via two‐way repeated‐measures ANOVAs. A between‐subject factor of sex, and a within‐subject factor of contraction time were applied to the participants' rating of fatigue, MVC, CV of torque, EMG rms, superimposed twitch, resting twitch, and VA data. Mauchly's test of sphericity was applied to the data, and non‐spherical data were subjected to Greenhouse‐Geisser corrections. If significant main or significant interaction effects were identified for each two‐way ANOVA, Tukey's multiple comparison post hoc tests were employed to examine how the sex of each group influenced the dependent variable at each time point of the contraction protocol. All data are presented as means ± the standard deviation of the mean. All statistical procedures were performed using IBM^®^ SPSS^®^ Statistics (version 22) with alpha levels set at <0.05.

## 3 RESULTS

### 3.1 Participant characteristics

Each group was well matched for age, body mass index, and limb preference. However, the mean height (*t*(6) = 3.64, *p* = 0.011), weight (*t*(6) = 2.63, *p* = 0.039), and length of the forearm applying force to the custom designed transducer (*t*(6) = 5.67, *p* = 0.001) were all greater for men compared to women (Table 1).

**TABLE 1:** Participant characteristics and baseline measurements

Variable	Males (mean ± *SD*)	Females (mean ± *SD*)
Number of participants	7	7
Age (years)	21.6 ± 1.1	22.0 ± 2.0
Age range (years)	21–24	20–25
Height (cm)	179.6 ± 4.5	167.7 ± 9.3 *
Weight (kg)	79.1 ± 12.8	63.4 ± 11.1 *
BMI	24.5 ± 3.5	22.4 ± 2.5
Limb preference (right/left)	7/0	7/0
Lever arm (cm)	24.2 ± 2.1	20.6 ± 2.0 *
MVC torque (*N*.m)	91.5 ± 8.5	41.4 ± 7.3 *
Torque coefficient of variation at 20% MVC (%)	2.06 ± 0.41	1.61 ± 0.38
Biceps brachii EMG rms at 20% MVC (mV)	0.11 ± 0.07	0.06 ± 0.01
Resting twitch (*N*.m)	14.4 ± 3.0	5.7 ± 1.8 *
Superimposed Twitch (*N*.m)	0.32 ± 0.18	0.19 ± 0.25
Voluntary Activation (%)	98.4 ± 1.4	98.2 ± 1.5

Note

Asterisk indicates a significant difference between male and female groups (*p* < 0.05). Baseline measurements are from the participants' first testing session.

Abbreviations: BMI, body mass index; EMG, electromyography; MVC, maximal voluntary contraction; *SD*, standard deviation.

### 3.2 Unfatigued muscle contractions

Baseline contraction data were obtained prior to undertaking the fatigue protocols. Given that similar outcomes were achieved in both testing sessions, baseline data are only reported here for the first testing session that each participant attended. No between‐group differences were identified for CV of torque, biceps EMG amplitude, superimposed twitch amplitude, or the level of voluntary activation. However, the male group had a significantly greater MVC torque (*t*(6) = 10.02, *p* < 0.001), and resting twitch amplitude (*t*(6) = 6.15, *p* < 0.001) compared to the female group (Table 1).

### 3.3 MVC during the fatiguing contractions

**FIGURE 2:**
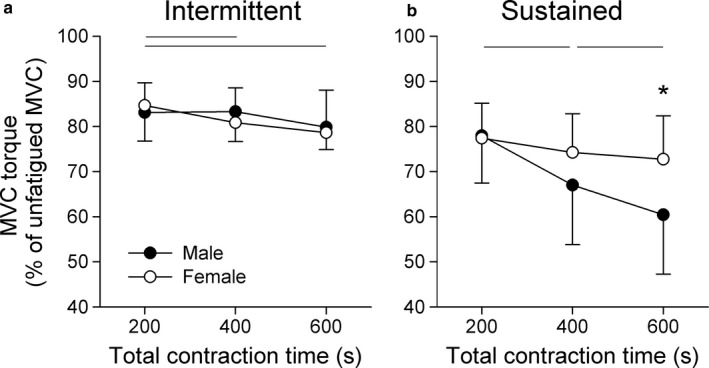
Maximal torque that males and females were able to generate throughout the intermittent (a) and sustained (b) isometric contraction protocols. Peak torque was measured during MVCs that were performed after the first, second, and third 200 s of contractions of each protocol. As MVC differed between groups for the baseline condition, MVCs obtained during the contraction protocol were normalized to unfatigued baseline data. Data are presented as the mean ± *SD* (*n* = 7). Horizontal lines indicate time effects (*p* < 0.05), and asterisk indicate group differences at that time point (*p* < 0.05)

The ability to perform maximal contractions was examined throughout each testing session, where MVC amplitude progressively declined regardless of the mode of contraction. For the intermittent mode of contraction, where 5 s of rest occurred with every 5 s of contraction, MVC declined by 16.9 ± 6.3% for men and 15.3 ± 5.0% for women following the initial 200 s of contraction. A main effect of time was detected for the intermittent contraction protocol (*F*(1.9, 11.8) = 19.93, *p* < 0.001), where post hoc analysis revealed that the 600 s MVC was significantly lower than the 200 s MVC (*p* = 0.003) and the 400 s MVC (*p* = 0.029, Figure 2a). There was no main effect of sex, or sex by time interaction, detected for MVC when performing sustained contractions.

Following the initial 200 s of sustained contraction, MVC declined from baseline levels by 22.0 ± 10.4% for the male group and 22.6 ± 7.8% for the female group. A main effect of time was identified for the sustained contraction protocol (*F*(1.4, 8.9) = 21.20, *p* = 0.001), where MVC declined from 200 to 400 s (*p* = 0.012), and 400 to 600 s (*p* = 0.047, Figure 2b). Although there was no main effect of sex for the sustained contractions, sex by time interaction occurred (*F*(1.7, 10.9) = 15.16, *p* < 0.001). The male group had a significantly larger decline in MVC after 600 s of contraction (*p* = 0.046), which indicates that sex‐related declines in MVC only emerged with the progression of the prolonged steady‐state contraction.

### 3.4 Elbow flexion torque CV (steadiness) and biceps brachii EMG during the fatiguing contractions

**FIGURE 3:**
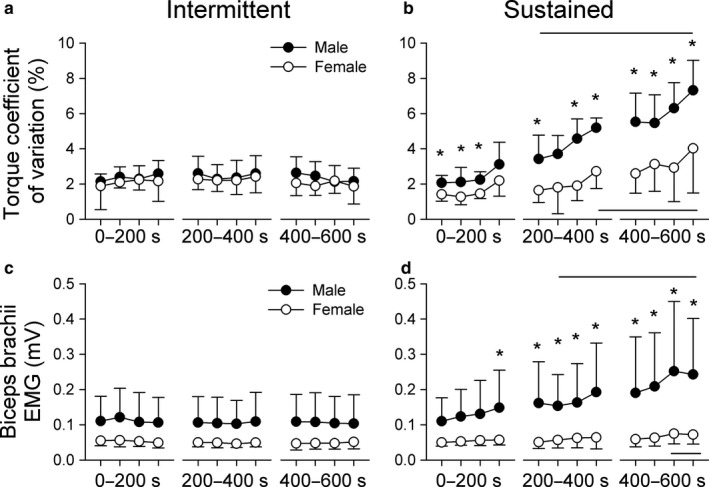
Elbow flexion steadiness was assessed by calculating the coefficient of variation of torque measured throughout the intermittent (a) and sustained (b) isometric contraction protocols. Coefficient of variation was calculated in 4 bins that spanned 200 s windows of the muscle contraction. The amplitude of biceps was calculated for the same regions throughout the intermittent (c) and sustained (d) isometric contraction protocols. All data are presented as the group mean ± *SD* (*n* = 7). Asterisks represent group differences at that time point (*p* < 0.05) and horizontal lines indicate data that were significantly greater than baseline measurements (*p* < 0.05)

Men and women were not significantly different at baseline for CV of torque and biceps brachii EMG amplitude (Table 1), a feature which did not change throughout the intermittent contraction protocol (Figure 3a and c). However, sex‐related differences emerged during the sustained contraction protocol. For torque CV there were main effects of sex (*F*(1,6) = 24.90, *p* = 0.002) and time (*F*(3.5, 21.5) = 21.17, *p* < 0.001), and a sex by time interaction (*F*(2.8, 15.9) = 3.52, *p* = 0.047). Post hoc analysis revealed that the male group had a greater CV than the female group from the onset of the sustained contraction, a difference which got progressively greater throughout the contraction (Figure 3b). Biceps EMG followed a similar pattern where there were main effects of sex (*F*(1,6) = 7.90, *p* = 0.031) and time (*F*(1.3, 8.2) = 6.42, *p* = 0.027), and a sex by time interaction (*F*(1.6, 9.7) = 5.36, *p* = 0.032). Post hoc analysis revealed that men and women had similar EMG relationships for the majority of the first 200 s of contraction, however, the male group was greater than the female group for the rest of the sustained contraction protocol (Figure 3c).

### 3.5 Rating of perceived fatigue

**FIGURE 4:**
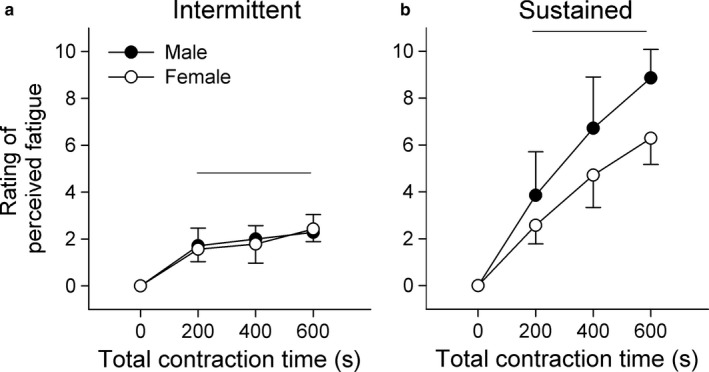
Rating of perceived fatigue for male and female participants during the intermittent (a) and sustained (b) elbow flexion contraction protocols. A rating of fatigue was collected immediately prior to the beginning of each contraction protocol (0 s). Subsequent measurements were obtained at the end of each 200 s contraction period. Data are presented as the mean ± *SD* (*n* = 7). A main effect of sex was only detected for the sustained contraction protocol. Horizontal lines indicate when perceived fatigue was significantly greater than baseline (*p* < 0.05)

Self‐perceived measures of fatigue were collected from each participant following each 200 s block of contraction. Similar to our quantitative fatigue measures, the intermittent protocol had no main effect of sex, but a main effect of time (*F*(2.1, 12.6) = 58.55, *p* < 0.001) where perceptions of fatigue during contractions were greater than baseline, and no interaction effect (Figure 4a). Unlike previous results, sex‐differences during the sustained contraction were not dependent on the duration of the contraction, as there was only a main effect of sex (*F*(1, 6) = 6.73, *p* = 0.041) and time (*F*(1.9, 11.6) = 169.9, *p* < 0.001) where perception of fatigue during contractions were greater than baseline (Figure 4b).

### 3.6 Superimposed twitch and resting twitch during the fatiguing contractions

**FIGURE 5:**
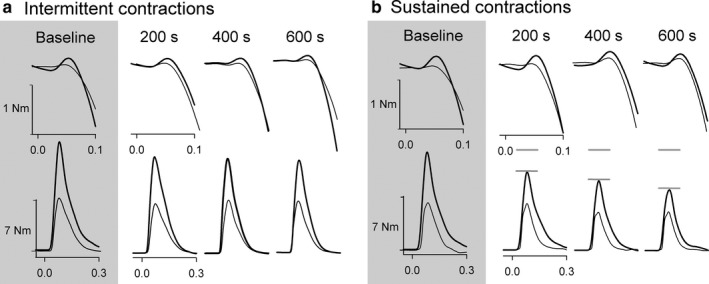
Representative torque associated with biceps brachii motor nerve stimulation during the intermittent (a) and sustained (b) elbow flexion contraction protocols. The top row in each panel represents the mean superimposed twitch at baseline, as well as the superimposed twitch following 200, 400, and 600 s of contraction. The lower panel represents the mean potentiated resting twitch collected during the same phases of the contraction protocol. Males are thicker lines whereas females are thinner lines. Zero seconds is the delivery of stimulation

**FIGURE 6:**
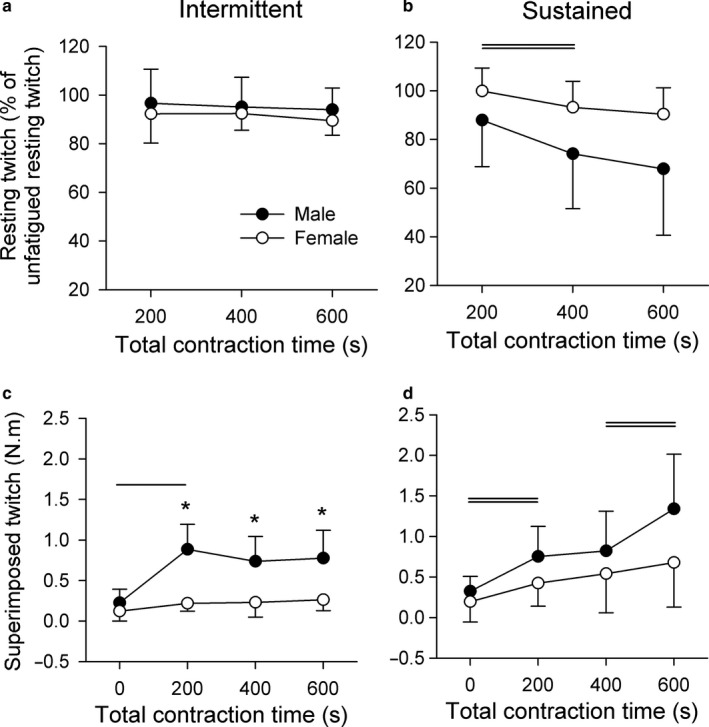
Resting twitch amplitude during the intermittent (a) and sustained (b) elbow flexion contraction protocols, as well as superimposed twitch amplitude during the intermittent (c) and sustained (d) elbow flexion contraction protocols. As resting twitch differed between groups for the baseline condition, resting twitches obtained during the contraction protocol are normalized to unfatigued baseline data. Twitch data are presented for males and females where data are presented as the mean ± *SD* (*n* = 7). Asterisks represent group differences at that time point (*p* < 0.05) and single horizontal lines indicate data that were significantly greater than baseline measurements for the sex by time interaction (*p* < 0.05). Double horizontal lines indicate post hoc results for main effect of time (*p* < 0.05)

Different twitch responses emerged between sexes with different modes of contraction (Figure 5a and b). Intermittent contractions elicited a main effect of sex (*F*(1,6) = 41.18, *p* = 0.001), time (*F*(2.4, 14.8) = 18.6, *p* < 0.001), and sex by time interaction (*F*(1.9, 11.4) = 5.77, *p* = 0.019). Post hoc analysis revealed that men increased superimposed twitch amplitude from baseline to the 200 s point, to which men were greater than women for the rest of the contraction protocol (Figure 6c). In contrast, sustained contractions only elicited a time difference (*F*(2.0, 12.3) = 12.98, *p* = 0.001), where twitch amplitude increased from baseline to 200 s, and from 400 to 600 s (Figure 6d).

Changes in resting twitch provide insight into the contractile properties of the muscle, where a reduction in twitch amplitude is typically associated with peripheral fatigue. Throughout the intermittent contraction protocol, there were no discernible differences between sexes, or changes in resting twitch amplitude over time (Figure 6a). However, for the sustained contractions, there was a main effect of sex (*F*(1, 6) = 6.21, *p* = 0.046) where the male group had reduced resting twitch amplitude during the protocol compared to female group (Figure 6b). A main effect of time (*F*(1.1, 7.0) = 7.10, *p* = 0.029) was also detected where resting twitch amplitude progressively declined over 600 s of sustained elbow flexion. Time‐dependent properties of the contracting muscle did not differ between sexes, as the time‐to‐peak and half‐relaxation times of the resting twitch were not different between sexes for either contraction protocol, or between fatigued and unfatigued muscle states (Table 2).

**TABLE 2:** Time‐to‐peak and half relation time for resting twitches collected during the intermittent and sustained contraction protocols

	Intermittent contractions				Sustained contractions			
	Baseline	200 s	400 s	600 s	Baseline	200 s	400 s	600 s
Time‐to‐peak (ms)								
Males	57 ± 9	53 ± 9	49 ± 4	49 ± 5	61 ± 15	49 ± 11	51 ± 13	48 ± 15
Females	49 ± 11	53 ± 7	49 ± 10	46 ± 10	53 ± 14	52 ± 8	55 ± 10	51 ± 9
Half‐relaxation time (ms)								
Males	47 ± 13	43 ± 10	44 ± 14	42 ± 14	49 ± 14	45 ± 13	39 ± 12	32 ± 14
Females	68 ± 28	70 ± 29	64 ± 27	59 ± 15	60 ± 11	55 ± 12	46 ± 7	41 ± 8

### 3.7 Level of voluntary activation

**FIGURE 7:**
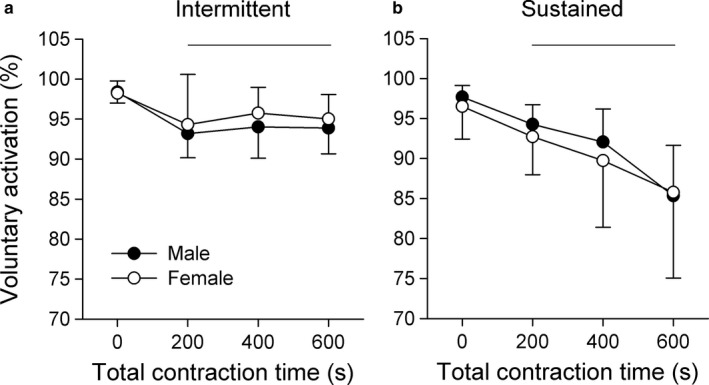
Voluntary activation for the male and female group during the intermittent (a) and sustained (b) contraction protocols. Voluntary activation was calculated for unfatigued baseline MVCs, and at the end of each 200 s contraction period. Data are presented as the mean ± *SD* (*n* = 7). A main effect of time was identified for both protocols, where horizontal lines indicate when voluntary activation was significantly lower than baseline (*p* < 0.05)

Impairments in voluntary activation is typically used as an indicator of central fatigue, as the voluntary activation calculation can explain declines in force output while accounting for peripheral fatigue that occurred during the voluntary contraction. For intermittent contractions, no sex‐related main or interaction effect was detected, but a main effect of time (*F*(1.3, 8.0) = 4.95, *p* = 0.049) was observed. Following the initial 200 s of contraction, voluntary activation declined by 5.2 ± 2.7% for men and 3.9 ± 6.0% for women, before remaining stable throughout the intermittent contraction protocol (Figure 7a). Sustained contractions elicited similar responses, where there were no sex‐related main or interaction effects, but a main effect of time (*F*(1.3, 8.0) = 17.31, *p* < 0.001) was observed. Voluntary activation declined by 3.4 ± 2.4% for the male group, and 3.8 ± 2.9% for the female group following the initial 200 s of sustained contraction, before progressive declining throughout the rest of the protocol (Figure 7b).

## 4 DISCUSSION

The purpose of this study was to examine fatigue‐related responses in a cohort of healthy young men and women during different modes of muscle contractions. Two elbow flexion protocols were performed, where participants performed sustained isometric contractions and intermittent isometric contractions in separate sessions. Overall, there were very few sex‐related differences in the fatigue responses identified for intermittent contractions. However, men exhibited enhanced muscle activity and decreased elbow flexion steadiness during sustained contractions, which was accompanied by a greater perception of fatigue.

### 4.1 Force generation during sustained isometric and intermittent contractions

One of the most fundamental indicators of motor fatigue is an inability to produce force in a maximally contracting muscle. In the current study, the amplitude of MVC was consistent at the end of each 200 s block of intermittent contraction. However, there was a progressive decline in MVC for the sustained contraction task for both the male and female groups. As such, exercise‐induced declines in maximal force generation were most prevalent when participants did not have the opportunity to relax the biceps brachii. This finding has been previously reported for the upper limb, where declines in force generation are more pronounced in submaximal sustained contractions compared to submaximal intermittent contractions (Adamo, Khodaee, Khodaee, Barringer, Johnson, & Martin, 2009). However, the current study builds upon this knowledge by illustrating that sex‐related differences influenced the ability to maintain steady contractions involving elbow flexors. In particular, biceps brachii activity increased at a greater rate in the men compared to the women during each 200 s block of sustained isometric contractions.

### 4.2 Elbow flexion steadiness differs between sexes during isometric contractions

In the current study, there were no sex‐related differences identified in unfatigued (baseline) elbow flexion steadiness, which contrasts to previous reports that men have greater steadiness than women over a range of contractions from 2.5% MVC to 75% MVC (Brown, Edwards, Edwards, & Jakobi, 2010). Interestingly, the female participants in the study of Brown et al. (2010) continued using oral contraceptives and were evaluated during the luteal phase of their menstrual cycle. Our female participants were not on oral contraception and were assessed during the early follicular phase of their menstrual cycle. Given that the oral contraception is associated with higher progesterone, estrogen, and basal core temperature (Minahan, Melnikoff, Melnikoff, Quinn, & Larsen, 2017), which is also associated with higher levels of unfatigued tremor (Tenan, Hackney, Hackney, & Griffin, 2016), it is possible that the combination of these factors indirectly influenced the unfatigued motor system to impair the ability to perform steady muscle contractions.

The submaximal contraction tasks caused different responses in elbow flexion steadiness between men and women. Notably, elbow flexion torque steadiness was relatively unaffected for the female group when each contraction protocol was performed, but the male group had progressively lower levels of steadiness during the prolonged isometric contractions. Although steadiness is a dimensionless measure where variability in torque is normalized to the torque amplitude (Kavanagh, Wedderburn‐Bisshop, Wedderburn‐Bisshop, & Keogh, 2016; Kenway et al., 2014), it cannot be discounted that enhanced strength in the male group caused some of the reductions in steadiness. In particular, numerical normalization does not account for the increased intramuscular pressure, increased mechanical occlusion of vasculature supplying the muscle, and decreased muscle perfusion potentially experienced by males when performing steady‐state contractions.

Fatigue‐related declines in steadiness for both groups aligned with the amplitude of biceps EMG, which suggests that the amount of drive to the target muscle played a critical role in steadiness responses in this study. Presumably, the increased muscle activity observed in males during the sustained contraction corresponds to additional recruitment of larger motor units and changes in motor unit firing rates to complete the task—both of which can alter torque steadiness. While the role that type III and IV afferents play in exercise performance has been elusive, several studies that have revealed different roles depending on whether contractions are unfatigued or fatigued (Amann et al., 2011; Amann, Proctor, Proctor, Sebranek, Pegelow, & Dempsey, 2009; Blain et al., 2016; Broxterman et al., 2018). In particular, group III/IV muscle afferents directly limit exercise performance to slow down the development of peripheral fatigue. However, with continued exercise the inhibiting role that group III/IV afferents play may be less effective during isometric contractions of a single muscle group (Broxterman et al., 2018), which is consistent with our finding of greater biceps brachii EMG for the male group during our isometric contraction task. An alternative proposition may lie in the actual muscles that are performing the task. In particular, there is some evidence to suggest that group III/IV muscle afferents may be excitatory to biceps motoneurones (Martin, Smith, Smith, Butler, Gandevia, & Taylor, 2006), however, it is unknown if this activity differs between sexes.

### 4.3 Central and peripheral mechanisms of muscle activation

Twitch interpolation was used at the end of each 200 s contraction block to reveal if central or peripheral fatigue processes differed between sexes during each contraction protocol. Our results indicate that voluntary activation, a measure that reflects the neural activation of muscle (Allen, Gandevia, Gandevia, & McKenzie, 1998; McKenzie, Bigland‐Ritchie, Gorman, & Gandevia, 1992; Todd, Taylor, & Gandevia, 2016), did not differ between men and women. This similarity between sexes was apparent during unfatigued maximal contractions as well as following fatiguing contractions. However, it should be noted that voluntary activation reflects changes in both the superimposed and resting twitch amplitude, and there were instances where the superimposed twitch for men was greater for both contraction protocols. As such, the regulation of force in each task may have been a dynamic process where the CNS adjusts its output depending on the nature of the task and the level of fatigue that is present.

Given that the resting twitch reflects the contractile mechanisms of the muscle (Allen et al., 1998; Todd, Taylor, Taylor, & Gandevia, 2003), and males had a greater baseline elbow flexion torque, it is not surprising that the male group had a larger unfatigued resting twitch compared to females. While it was hypothesized that men would be more fatigable than women during the sustained contraction protocols, the magnitude of this effect during the initial stages of the protocol was unexpected. After 200 s of sustained contraction, the male group had a 12% reduction in resting twitch amplitude which far exceeds the 1% reduction in resting twitch amplitude for the female group (Figure 6b). As such, the female muscle was more resistant to fatigue during the initial stages of steady‐state contractions, which is consistent with studies that have identify fatigue resistance in females during isometric and slow, dynamic contraction tasks, and recovery from these contraction tasks (Hunter, Butler, et al., 2006; Senefeld et al., 2018; Yoon et al., 2015).

A potential mechanism of sex‐dependent fatigue resistance may lie in Calcium ion handling, as female muscle exhibits lower Ca^2+^‐ATPase activity in unfatigued muscle, but preserves Ca^2+^‐ATPase activity with high‐intensity exercise (Harmer et al., 2014). Our findings of enhanced peripheral fatigue in the male group (decreased resting twitch amplitude) is also consistent with previous reports of upper limb fatigue (Hunter, Butler, et al., 2006). However, unlike other investigations, we did not identify sex‐related differences in the time‐to‐peak and half relaxation time of resting twitches (Hunter, Butler, et al., 2006; Wust, Morse, Morse, Haan, Jones, & Degens, 2008). As such, it appears that fiber type composition, and properties associated with muscle relaxation such as Ca^2+^ uptake to the sarcoplasmic reticulum, Ca^2+^dissociation from troponin, and cessation of cross‐bridge cycling (Allen, Lamb, Lamb, & Westerblad, 2008; Neyroud et al., 2016), may have been similar between males and females.

### 4.4 Self‐perception of fatigue is higher in males during isometric fatiguing contractions

While the relationship between perception of fatigue and quantitative measures of fatigue has not been clarified, there are reports of linearity between the Borg Category‐Ratio scale and the intensity of isometric contractions involving the flexor pollicis longus (Whittaker, Sonne, Sonne, & Potvin, 2019), quadriceps (Pincivero, Coelho, Coelho, & Erikson, 2000), and elbow flexors (Kavanagh, McFarland, McFarland, & Taylor, 2019). Moreover, sex‐differences in perceived exertion may be most evident for isometric contractions, as perceptions of effort during dynamic contractions do not typically differ between men and women (Hunter, 2009; Springer & Pincivero, 2010). This viewpoint is consistent with the results of the current study, as the timing of declines in force in the male group coincided with a greater perception of fatigue compared to the female group. In the context of our voluntary activation results, these sex‐differences in perceived fatigue were somewhat unexpected. Voluntary activation is often associated with perceptions of effort (Smith, Martin, Martin, Gandevia, & Taylor, 2007; Søgaard, Gandevia, Gandevia, Todd, Petersen, & Taylor, 2006), however, our results indicate that the relationship between perception of fatigue and voluntary activation may differ with sex and mode of contraction. Overall, it appears that caution should be used if trying to attribute the perception of effort or fatigue with the ability to voluntarily activate muscle—particularly when the test sample includes men and women.

### 4.5 Factors underlying fatigue during sustained isometric contractions

Our interpretation of results assumes that the isometric contraction invoked blood flow changes in the muscle, and that these changes in the muscle were exacerbated with the prolonged contraction protocol. However, this perspective is not without limitations. In particular, larger muscle mass in the male group would presumably be accompanied by a larger blood supply. Thus, a proportional blood supply between sexes may also lead to proportional levels of occlusion. There is also evidence to suggest that larger absolute levels of force generation in males may cause greater occlusion in blood supply in this group (Hunter, Schletty, et al., 2006). However this is not a simple issue because studies that match men and women for strength have shown greater fatigue in men than women for the upper limb during intermittent contractions (Fulco et al., 1999; Hunter et al., 2004a, 2004b) but not during static contractions (Hunter et al., 2004a, 2004b). Although occlusion is often linked to increased concentrations of intramuscular hydrogen ion and peripheral fatigue, the effects of reduced pH on isometric contractions have been shown to be temperature‐dependent in animal preparations (Pate, Bhimani, Bhimani, Franks‐Skiba, & Cooke, 1995; Wiseman, Beck, Beck, & Chase, 1996). As such, cellular mechanisms associated with pH changes may only have a minor role in enhancing fatigue under physiological temperatures in humans.

### 4.6 Considerations

There are features of our participant pool that must be considered when interpreting our data. Although, subject numbers in each group were low, clear and consistent statistical differences were identified across the dependent variables assessed in the study. Importantly, the significant differences observed between sexes and the enhanced peripheral fatigue for the male group during sustained isometric contractions (a) align with several theories of sex‐related differences in motor performance, and (b) are comparable with other experimental studies. A further consideration is that the training status of the groups was not controlled. Both groups were recreationally active, but it is possible that the two‐fold increase in male MVC compared to female MVC could be a factor of different training histories. However, it should be noted that neural activation of muscle is task‐specific (Sekiguchi, Nakazawa, Nakazawa, & Hortobágyi, 2013; Uematsu, Sekiguchi, Sekiguchi, Kobayashi, Hortobágyi, & Suzuki, 2011), and the greatest improvements in performance can occur when task‐specific training has taken place (Hirayama, Yanai, Yanai, Kanehisa, Fukunaga, & Kawakami, 2012). Informal questioning indicated that no participant in our study had a training history involving isometric contractions, which suggests that a history of performing isometric elbow flexions did not play a role in our findings. Finally, given that the baseline MVC amplitude was different between males and females, it cannot be ruled out that group‐differences in torque steadiness and muscle activation were due sex‐differences in strength. However, this will typically be a limitation in applied research into sex differences as men are typically stronger than women in upper limb muscles. Future research may consider strength‐matching males and females in accordance with the strength‐matching recruitment and testing protocols of Hunter and colleagues (Hunter et al., 2004a, 2004b; Hunter, Schletty, et al., 2006).

### 4.7 Conclusions

This study identified very few sex‐related differences in the fatigue responses identified for intermittent isometric elbow flexions. However, men exhibited enhanced muscle activity and decreased elbow flexion steadiness during sustained isometric elbow flexions. The origin of the mechanism underlying these fatigue responses had a muscle component. This was evident from a lack of sex differences in voluntary activation of the biceps brachii, combined with sex‐related differences in the resting twitch amplitude for the fatigued muscle during sustained contractions. Presumably, muscle‐related differences contributed to altered sensory feedback to the CNS, as the enhanced fatigue observed for men during the sustained isometric contraction was accompanied by a greater perception of fatigue. Overall, it appears that both men and women are similarly resistant to fatigue when performing low‐intensity intermittent contractions. However, in the absence of relaxation phases, males are less resistant to fatigue during low‐intensity sustained contractions.

## CONFLICT OF INTERESTS

None of the authors have potential conflicts of interest to be disclosed.

## AUTHORS' CONTRIBUTIONS

Experiments were performed in the Neural Control of Movement Laboratory at Griffith University. All authors designed the study protocol. KAS and JJK. acquired and analyzed the data. All authors interpreted the data and drafted or revised the final manuscript. All authors approved the final version of the manuscript and agree to be accountable for all aspects of the work in ensuring that questions related to the accuracy or integrity of any part of the work are appropriately investigated and resolved. All persons designated as authors qualify for authorship, and all those who qualify for authorship are listed.
